# Association of *IL16* rs11556218 and *IL1B* rs16944 polymorphisms with type 2 diabetes in the Kinh Vietnamese population

**DOI:** 10.1371/journal.pone.0358570

**Published:** 2026-09-28

**Authors:** Quynh Ai Lam, Hen Huu Phan, Linh Hoang Gia Le, Minh Duc Do

**Affiliations:** 1 Department of Physiology-Pathophysiology-Immunology-Pharmacology, School of Medicine, University of Medicine and Pharmacy at Ho Chi Minh City, Ho Chi Minh City, Vietnam; 2 Department of Endocrinology, Cho Ray Hospital, Ho Chi Minh City, Vietnam; 3 Center for Molecular Biomedicine, University of Medicine and Pharmacy at Ho Chi Minh City, Ho Chi Minh City, Vietnam; Neyshabur University of Medical Sciences, IRAN, ISLAMIC REPUBLIC OF

## Abstract

**Background:**

Type 2 diabetes mellitus (T2DM) is a multifactorial metabolic disease in which inflammatory mechanisms are involved in disease development and progression. Polymorphisms in cytokine-related genes may influence susceptibility to T2DM, yet evidence in Vietnamese individuals remains scarce. This study was designed to investigate whether *IL16* rs11556218 and *IL1B* rs16944 are associated with T2DM in Kinh Vietnamese adults.

**Methods:**

An analytical cross-sectional unmatched case-control study was conducted in 392 unrelated Kinh Vietnamese individuals, including 196 patients with T2DM and 196 non-diabetic controls, recruited at Cho Ray Hospital, Ho Chi Minh City, between February and August 2025. Peripheral blood samples were collected for genomic DNA extraction. Genotyping of *IL16* rs11556218 and *IL1B* rs16944 was performed using TaqMan SNP Genotyping Assays. Hardy-Weinberg equilibrium was assessed in the control group. Associations between the investigated polymorphisms and T2DM were analyzed under codominant, dominant, recessive, and log-additive models, with adjustment for body mass index (BMI), waist-to-hip ratio (WHR), and family history of diabetes.

**Results:**

Both polymorphisms satisfied the Hardy-Weinberg equilibrium in the control group. For *IL16* rs11556218, significant differences in allele and genotype frequencies were observed between patients and controls, and the G allele showed a nominal association with lower odds of T2DM. After adjustment for BMI, WHR, and family history of diabetes, the TG genotype showed a nominal association with lower odds of T2DM in the codominant model (adjusted OR = 0.56, 95% CI: 0.32–0.98; nominal P = 0.026). Nominal associations were also observed in the dominant model (adjusted OR = 0.52, 95% CI: 0.30–0.89; nominal P = 0.016) and the log-additive model (adjusted OR = 0.52, 95% CI: 0.32–0.85; nominal P = 0.0081). For *IL1B* rs16944, genotype distribution likewise differed between the two groups. Under the recessive model, the CC genotype showed a nominal association with higher odds of T2DM after adjustment (adjusted OR = 2.13, 95% CI: 1.20–3.79; nominal P = 0.0089). None of these associations met the Bonferroni-corrected significance threshold of P < 0.00625. Compared with controls, participants with T2DM had higher BMI and WHR, higher triglyceride levels, lower HDL cholesterol, higher fasting plasma glucose and blood pressure, and more commonly reported a family history of diabetes.

**Conclusions:**

*IL16* rs11556218 and *IL1B* rs16944 showed nominal associations with T2DM in this sample of Kinh Vietnamese adults, although none remained statistically significant after Bonferroni correction. These exploratory findings require confirmation in larger independent studies before any firm conclusions regarding their biological or clinical relevance can be drawn.

## Introduction

Type 2 diabetes mellitus (T2DM) is a multifactorial metabolic disease characterized by insulin resistance and progressive pancreatic β-cell dysfunction. It accounts for most diabetes cases worldwide. Over recent decades, its prevalence has risen markedly, and both cardiovascular and microvascular complications remain major contributors to morbidity and mortality [1]. In Vietnam, about 2.5 million adults aged 20–79 years were living with diabetes in 2024, corresponding to an age-standardised prevalence of 3.4%, and this burden is projected to increase further by 2050 [1].

Asian populations tend to develop T2DM at younger ages and at lower BMI than White European populations. This phenotype may reflect greater insulin resistance at relatively low BMI, earlier β-cell dysfunction, increased visceral adiposity, and population-specific genetic susceptibility [2–5]. Although many genetic studies have focused on East and South Asian populations, data from Southeast Asia remain limited [6–8]. The Kinh ethnic group, which constitutes the majority of the Vietnamese population, has rarely been included in genetic studies of T2DM [9–11]. Further studies in this population may help clarify genetic factors contributing to T2DM susceptibility.

In addition to lifestyle and environmental determinants, chronic low-grade inflammation is now considered an important contributor to the pathophysiology of T2DM. Pro-inflammatory cytokines have been shown to induce β-cell stress, interfere with insulin signaling, and disturb metabolic homeostasis [12–14]. Among the cytokines implicated in these processes, interleukin-1β (IL-1β) and interleukin-16 (IL-16) are of particular interest because they represent biologically plausible candidates acting through partly distinct inflammatory pathways. Accordingly, genetic variation affecting these cytokine pathways may partly explain interindividual differences in T2DM susceptibility [15–18].

IL-1β, encoded by *IL1B*, is a key mediator of inflammatory responses, inflammasome activation, and insulin resistance, all of which are central features of T2DM pathogenesis [19]. The promoter polymorphism rs16944 has been suggested to influence transcriptional regulation and cytokine production [20–22]. This variant has been examined in relation to T2DM and related metabolic traits in several studies and meta-analyses, particularly in Asian populations; however, the available findings remain inconsistent. Some studies have reported a significant positive association with T2DM, whereas others have not demonstrated a clear relationship, possibly because of differences in genetic background, environmental context, or study methodology [20,21,23].

IL-16 is an immunomodulatory cytokine that binds to CD4 and promotes T-cell recruitment and activation, thereby enhancing inflammatory signaling pathways involved in insulin resistance and metabolic dysfunction [24,25]. Increased circulating IL-16 concentrations have been described in obesity, and this cytokine may participate in adipogenesis, inflammatory responses, and glucose-lipid metabolism [25]. The missense variant rs11556218 may modify protein structure or biological function and has been investigated in relation to T2DM and adverse metabolic traits in several ethnic populations, including Egyptian and Han Chinese groups, although the evidence remains limited and not entirely consistent [16,18]. To date, this locus has not, to our knowledge, been studied in Vietnamese individuals.

Previous genetic studies of T2DM in the Kinh Vietnamese population have mainly concentrated on loci associated with insulin secretion or adipokine regulation, including KCNJ11, ABCC8, and ADIPOQ, and these studies have reported associations with T2DM and metabolic syndrome. By comparison, inflammation-related genetic variants have received far less attention [9–11]. Therefore, this study aimed to investigate the associations of *IL1B* rs16944 and *IL16* rs11556218 with T2DM in Kinh Vietnamese individuals.

## Materials and methods

### Study design and participants

This analytical cross-sectional hospital-based unmatched case-control study was conducted at the Outpatient Department of Cho Ray Hospital, a tertiary referral hospital in Ho Chi Minh City, between February and August 2025. Using convenience sampling, a total of 392 unrelated Kinh Vietnamese individuals were recruited, including 196 participants with T2DM and 196 individuals without T2DM. Cases and controls were not individually matched based on age, sex, or other characteristics.

The diagnosis of T2DM was established in accordance with the American Diabetes Association 2025 criteria [26]. Individuals in the non-T2DM group had no previous diagnosis of diabetes and had fasting plasma glucose levels below 100 mg/dL at the time of enrollment. Participants were excluded if they had type 1 diabetes, gestational diabetes, liver dysfunction defined by alanine aminotransferase (ALT) and/or aspartate aminotransferase (AST) levels exceeding three times the upper limit of normal, a prior diagnosis of autoimmune disease, use of medications known to affect blood glucose levels, or refusal to participate. Written informed consent was obtained from all participants before enrollment.

### Clinical and biochemical assessment

Clinical and demographic information was collected using a structured case record form. The study variables included age at diagnosis (for participants with T2DM), age at recruitment (for controls), sex, family history of diabetes, BMI, waist circumference, hip circumference, waist-to-hip ratio (WHR), systolic and diastolic blood pressure, fasting plasma glucose, HbA1c (T2DM group only), triglycerides, total cholesterol, high-density lipoprotein (HDL) cholesterol, low-density lipoprotein (LDL) cholesterol, and serum creatinine.

Anthropometric measurements were obtained with participants barefoot and dressed in light clothing. BMI was calculated as weight in kilograms divided by height in meters squared (kg/m²). Waist circumference was measured at the midpoint between the lower costal margin and the iliac crest, whereas hip circumference was measured at the widest level of the buttocks. WHR was derived by dividing waist circumference by hip circumference. Blood pressure was measured after 5–10 minutes of rest using a standardized aneroid sphygmomanometer. Two readings were recorded at an interval of 1–2 minutes. When the difference between the two measurements exceeded 10 mmHg, a third reading was obtained, and the mean of the latter two values was used for analysis.

Following an overnight fast of at least 8 hours, venous blood samples were collected at the Department of Biochemistry, Cho Ray Hospital. HbA1c was measured only in participants with T2DM and was not available for the control group. It was measured in EDTA-anticoagulated whole blood by high-performance liquid chromatography using an automated Tosoh HLC-723G8 analyzer. Fasting plasma glucose, triglycerides, total cholesterol, HDL cholesterol, LDL cholesterol, and serum creatinine were analyzed on an ADVIA 1800 system (Siemens) with reagents supplied by the manufacturer. Internal and external quality control procedures were performed in accordance with laboratory regulations.

### Sample size estimation

Sample size was calculated using the Genetic Power Calculator [27] based on previously reported allele frequencies [28] and genotype relative risks for rs16944 [29] and rs11556218 [16]. Under a multiplicative genetic model, assuming a type I error rate of 0.05 and statistical power of 80%, the estimated minimum sample size was 139 participants per group for rs16944 and 157 participants per group for rs11556218. To ensure adequate statistical power, the final sample included 196 participants with T2DM and 196 participants without T2DM.

### DNA extraction and genotyping

Peripheral venous blood (2–3 mL) was collected into EDTA tubes for genomic analysis. Genomic DNA was extracted from whole blood using the QIAamp DNA Mini Kit (QIAGEN, Hilden, Germany) according to the manufacturer’s instructions. DNA concentration and purity were assessed using a NanoDrop 2000/2000C spectrophotometer (Thermo Fisher Scientific, USA), and DNA was stored at −30°C until genotyping.

Genotyping of *IL1B* rs16944 and *IL16* rs11556218 was performed using TaqMan SNP Genotyping Assays on a QuantStudio™ 5 Real-Time PCR System (Thermo Fisher Scientific, USA). All DNA samples collected in this study were diluted to a final concentration of approximately 5 ng/µL. For each SNP, the real-time PCR reaction was performed in a total volume of 25 µL, containing 12.5 µL of TaqMan Genotyping Master Mix (Thermo Fisher Scientific, cat. no. 4371353), 1.25 µL of the corresponding 40X TaqMan SNP Genotyping Assay (assay ID C_1839943_10 for *IL1B* rs16944 or C_25646461_40 for *IL16* rs11556218), an appropriate volume of diluted genomic DNA template, and nuclease-free water to a final volume of 25 µL. The thermal profile included a pre-read step at 60°C for 30 seconds, an initial denaturation at 95°C for 10 minutes, followed by 40 cycles of 95°C for 15 seconds and 60°C for 1 minute, and a final post-read step at 60°C for 30 seconds. Each run included a negative control (ddH_2_O) to monitor contamination and a positive control previously confirmed by Sanger sequencing to ensure genotyping accuracy. Genotyping was based on allele-specific TaqMan minor groove binder (MGB) probes in a real-time PCR assay. For *IL1B* rs16944, the VIC-labeled probe detected the C allele and the FAM-labeled probe detected the T allele. For *IL16* rs11556218, the VIC-labeled probe detected the T allele, whereas the FAM-labeled probe detected the G allele. Genotypes were assigned according to fluorescence patterns, with single-dye signals indicating homozygosity for the corresponding allele and dual-dye signals indicating heterozygosity.

### Statistical analysis

Data were entered into EpiData version 3.1 and analyzed using SPSS version 26.0 and SNPStats. Continuous variables were presented as mean ± standard deviation or median with interquartile range, depending on data distribution, whereas categorical variables were reported as counts and percentages. Differences between the T2DM and control groups were assessed using Student’s t-test or the Mann-Whitney U test for continuous variables and the chi-square test or Fisher’s exact test for categorical variables, as appropriate. Hardy-Weinberg equilibrium for each polymorphism was examined in the control group by the exact test. The associations between each polymorphism and T2DM were assessed in SNPStats under codominant, dominant, recessive, and log-additive genetic models, with odds ratios (ORs) and 95% confidence intervals (CIs) reported. Adjusted analyses were performed in SNPStats, controlling for BMI, WHR, and family history of diabetes. BMI and WHR were both retained because they reflect different aspects of adiposity: BMI reflects overall adiposity, whereas WHR reflects abdominal fat distribution. Potential multicollinearity between BMI and WHR was assessed using the variance inflation factor (VIF), with values below 2 considered acceptable. All tests were two-sided. To account for multiple testing, a Bonferroni correction was applied to the eight primary adjusted association tests arising from the evaluation of two polymorphisms under four genetic models, resulting in a corrected significance threshold of P < 0.00625 (0.05/8). Associations with nominal P values <0.05 that did not meet the corrected threshold were considered exploratory.

### Ethics approval

The study protocol was reviewed and approved by the Ethics Committee of Cho Ray Hospital (No. 1886/CN-HDDD). All study procedures were conducted in accordance with the Declaration of Helsinki. Written informed consent was obtained from each participant before enrollment.

## Results

### Study population characteristics

The study included 392 Kinh Vietnamese participants, consisting of 196 patients with T2DM and 196 controls. Sex distribution was similar between the two groups (P = 0.297). Mean age at diagnosis in the T2DM group was comparable to mean age at recruitment in the control group (51.20 ± 11.27 vs. 51.05 ± 11.47 years, P = 0.891). Among patients with T2DM, median disease duration was 10.0 years (IQR: 4.6–15.8).

A family history of diabetes was reported more frequently in the T2DM group than in controls (61.2% vs. 25.5%, P < 0.001). Participants with T2DM also had higher BMI, WHR, fasting plasma glucose, systolic blood pressure, diastolic blood pressure, triglycerides, and serum creatinine. In contrast, total cholesterol, HDL cholesterol, and LDL cholesterol were lower in the T2DM group. All of these differences were statistically significant (Table 1).

**Table 1:** Baseline clinical and biochemical characteristics of the studied population.

Characteristics	Studied population	T2DM N = 196	Controls N = 196	P value
Males/females	148/244	79/117	69/127	0.297
Duration of diabetes (yr)	–	10.0 (4.6-15.8)	–	n/a
Age at diagnosis (T2DM)/age at recruitment (Controls)	51.13 ± 11.36	51.20 ± 11.27	51.05 ± 11.47	0.891
Family history of diabetes n (%)	170 (43.4)	120 (61.2)	50 (25.5)	<0.001*
BMI (kg/m2)	23.4 (21.4-25.7)	24.0 (22.1-26.4)	23.1 (21.1-25.0)	<0.001*
WHR	0.892 ± 0.079	0.928 ± 0.068	0.857 ± 0.072	<0.001*
SBP (mmHg)	130.0 (115.0-140.0)	131.3 (120.0-150.0)	125.0 (110.0-135.0)	<0.001*
DBP (mmHg)	80.0 (70.0-84.4)	80.0 (70.0−80.0)	80.0 (70.0-85.0)	0.003*
HbA1c (%)	–	7.65 (6.71-8.98)	–	n/a
FPG (mg/dL)	97.0 (89.3-131.0)	131.0 (111.0-168.8)	91.0 (86.0-95.0)	<0.001*
Triglycerides (mg/dL)	153.5 (109.0-233.5)	166.5 (126.0-263.0)	138.0 (92.0-202.0)	<0.001*
Total cholesterol (mg/dL)	186.0 (156.0-223.0)	175.0 (140.0-214.0)	196.0 (173.0-226.0)	<0.001*
HDL cholesterol (mg/dL)	46.0 (38.0-55.8)	44.5 (37.0-54.0)	48.0 (40.0-57.0)	0.006*
LDL cholesterol (mg/dL)	111.3 (83.8-146.4)	95.4 (73.3-124.9)	127.1 (104.2-159.6)	<0.001*
Serum creatinine (mg/dL)	0.79 (0.66-0.99)	0.86 (0.69-1.10)	0.76 (0.65-0.91)	<0.001*

BMI = body mass index, DBP = diastolic blood pressure, FPG = fasting plasma glucose, HDL = high-density lipoprotein, LDL = low-density lipoprotein, SBP = systolic blood pressure, T2DM = type 2 diabetes mellitus, WHR = waist-hip ratio.

Data are presented as mean ± standard deviation, median (interquartile range), or n (%), as appropriate.

* Statistically significant.

### Genotype distributions and Hardy-Weinberg equilibrium

In the control group, genotype distributions for both polymorphisms were consistent with the Hardy-Weinberg equilibrium. Exact-test P values were 0.63 for *IL16* rs11556218 and 0.15 for *IL1B* rs16944 (Table 2).

**Table 2:** Allele and genotype frequencies of rs11556218 and rs16944.

SNP	Allele/ Genotype	T2DM n (%)	Controls n (%)	P value	HWE P value (controls)
rs11556218	T	345 (88.0)	320 (81.6)	0.013*	–
	G	47 (12.0)	72 (18.4)		
	T/T	152 (77.6)	129 (65.8)	0.031*	0.63
	T/G	41 (20.9)	62 (31.6)		
	G/G	3 (1.5)	5 (2.6)		
rs16944	T	185 (47.2)	210 (53.6)	0.074	–
	C	207 (52.8)	182 (46.4)		
	T/T	47 (24.0)	51 (26.0)	0.044*	0.15
	T/C	91 (46.4)	108 (55.1)		
	C/C	58 (29.6)	37 (18.9)		

* Statistically significant.

MAF = 0.18 (G allele).

MAF = 0.46 (C allele).

HWE, Hardy–Weinberg equilibrium. HWE was assessed in the control group using an exact test.

### Association analyses for *IL16* rs11556218

For *IL16* rs11556218, both allele and genotype frequencies differed significantly between cases and controls. The T allele was more common in the T2DM group, whereas the G allele was less frequent (P = 0.013). Genotype distribution also differed between groups (P = 0.031). The TT genotype was observed in 77.6% of patients with T2DM and 65.8% of controls, while the TG genotype was more frequent in controls than in cases (31.6% vs. 20.9%). The minor allele frequency of G was 0.18 (Table 2). In unadjusted analyses, nominal associations were identified under the codominant, dominant, and log-additive models. Using TT as the reference, the TG genotype showed a nominal association with lower odds of T2DM (OR = 0.56, 95% CI: 0.35–0.88), whereas the GG genotype was not significantly associated with disease risk (OR = 0.51, 95% CI: 0.12–2.17). When TG and GG were combined, carriers of the G allele had lower odds of T2DM than TT homozygotes (OR = 0.56, 95% CI: 0.36–0.87; P = 0.0097). A similar finding was obtained under the log-additive model (OR = 0.60, 95% CI: 0.40–0.89; P = 0.012). No nominal association was observed under the recessive model (P = 0.47) (Table 3).

**Table 3:** Association of rs11556218 with T2DM (crude and adjusted analyses).

Model	Genotype	T2DM (n)	Controls (n)	Crude OR (95% CI)	P value	Adjusted OR† (95% CI)	P value^†^
Codominant	T/T	152	129	1	0.035*	1	0.026*
	T/G	41	62	**0.56 (0.35-0.88)**		**0.56 (0.32-0.98)**	
	G/G	3	5	0.51 (0.12-2.17)		0.18 (0.03-1.06)	
Dominant	T/T	152	129	1	0.0097*	1	0.016*
	T/G-G/G	44	67	**0.56 (0.36-0.87)**		**0.52 (0.30-0.89)**	
Recessive	T/T-T/G	193	191	1	0.47	1	0.081
	G/G	3	5	0.60 (0.14-2.50)		0.21 (0.04-1.23)	
Log-additive	–	–	–	**0.60 (0.40-0.89)**	0.012*	**0.52 (0.32-0.85)**	0.0081*

N = 392.

^†^Adjusted for BMI, WHR, and family history of diabetes.

*Nominal P < 0.05. Bonferroni correction was applied to the eight primary adjusted association tests, with statistical significance defined as P < 0.00625; no adjusted association met the corrected threshold.

Adjustment for BMI, WHR, and family history of diabetes yielded largely similar results. In the codominant model, the TG genotype showed a nominal association with reduced odds of T2DM relative to TT (adjusted OR = 0.56, 95% CI: 0.32–0.98), whereas the GG genotype remained non-significant (adjusted OR = 0.18, 95% CI: 0.03–1.06); the overall nominal P value for the codominant model was 0.026. Under the dominant model, the combined TG and GG genotypes showed a nominal association with lower odds of T2DM than TT (adjusted OR = 0.52, 95% CI: 0.30–0.89; nominal P = 0.016). The log-additive model also showed a nominal association after adjustment (adjusted OR = 0.52, 95% CI: 0.32–0.85; nominal P = 0.0081). No association was observed under the recessive model (P = 0.081). None of the nominal associations met the Bonferroni-corrected significance threshold of P < 0.00625 (Table 3).

### Association analyses for *IL1B* rs16944

The pattern for *IL1B* rs16944 differed from that observed for *IL16* rs11556218. Allele frequencies did not differ significantly between groups (P = 0.074), whereas genotype distribution showed a significant between-group difference (P = 0.044). The CC genotype was more frequent in participants with T2DM than in controls (29.6% vs. 18.9%), while the TC genotype was more common in controls (55.1% vs. 46.4%). The minor allele frequency of C was 0.46 (Table 2). In the crude analysis, evidence of association was mainly confined to the recessive model. Individuals with the CC genotype had higher odds of T2DM than those with TT or TC genotypes combined (OR = 1.82, 95% CI: 1.12–2.86; P = 0.013). In the codominant model, the OR for CC relative to TT was elevated, although its 95% CI included 1 (OR = 1.69, 95% CI: 0.96–3.03), whereas the TC genotype did not differ significantly from TT. Neither the dominant model nor the log-additive model reached statistical significance (Table 4). After adjustment for BMI, WHR, and family history of diabetes, the recessive model showed a nominal association, with an adjusted OR of 2.13 (95% CI: 1.20–3.79; nominal P = 0.0089). The overall codominant model also yielded a nominal P value of 0.022, although the genotype-specific 95% CIs for TC and CC relative to TT included 1. Neither the dominant model nor the log-additive model showed evidence of association after adjustment. Neither the recessive nor the codominant finding met the Bonferroni-corrected significance threshold (Table 4).

**Table 4:** Association of rs16944 with T2DM (crude and adjusted analyses).

Model	Genotype	T2DM (n)	Controls (n)	Crude OR (95% CI)	P value	Adjusted OR† (95% CI)	P value^†^
Codominant	T/T	47	51	1	0.043*	1	0.022*
	T/C	91	108	0.92 (0.56-1.49)		0.76 (0.42-1.37)	
	C/C	58	37	1.69 (0.96-3.03)		1.78 (0.89-3.56)	
Dominant	T/T	47	51	1	0.64	1	0.99
	T/C-C/C	149	145	1.11 (0.70-1.75)		1.00 (0.57-1.73)	
Recessive	T/T-T/C	138	159	1	0.013*	1	0.0089*
	C/C	58	37	**1.82 (1.12-2.86)**		**2.13 (1.20-3.79)**	
Log-additive	–	–	–	1.30 (0.98-1.72)	0.072	1.32 (0.94-1.86)	0.11

N = 392.

^†^Adjusted for BMI, WHR, and family history of diabetes.

*Nominal P < 0.05. Bonferroni correction was applied to the eight primary adjusted association tests, with statistical significance defined as P < 0.00625; no adjusted association met the corrected threshold.

Assessment of collinearity for BMI and WHR showed tolerance values of 0.719 and VIF values of 1.39 for both variables, indicating no meaningful multicollinearity (Table 5).

**Table 5:** Assessment of multicollinearity between BMI and WHR.

Variable	Tolerance	VIF
BMI	0.719	1.39
WHR	0.719	1.39

## Discussion

In this analytical cross-sectional hospital-based unmatched case-control study of Kinh Vietnamese adults, *IL16* rs11556218 and *IL1B* rs16944 showed nominal associations with T2DM, although their association patterns differed. After adjustment for BMI, WHR, and family history of diabetes, nominal associations were observed for rs11556218 under the codominant, dominant, and log-additive models and for rs16944 under the recessive model. However, none of these associations met the Bonferroni-corrected significance threshold. These exploratory findings suggest that variation in inflammation-related genes may be related to T2DM susceptibility in this population, which is consistent with current evidence implicating chronic low-grade inflammation in insulin resistance and β-cell dysfunction [30,31].

For *IL16* rs11556218, the G allele showed a nominal association with lower odds of T2DM in our study population. This finding differs from reports in some other ethnic groups, in which the same variant has been associated with increased diabetes risk or adverse metabolic traits related to T2DM [16,18]. Such variation across studies is not surprising in a complex disorder such as T2DM, in which the effect of a single polymorphism may depend on ethnic background, linkage disequilibrium structure, environmental influences, and the broader metabolic setting. Given the established role of IL-16 in leukocyte recruitment and immune regulation, together with growing evidence of its involvement in chronic inflammatory conditions, it is biologically plausible that rs11556218 may be related to interindividual differences in inflammatory response [24,25]. However, the present study was not designed to determine the functional consequences of this variant, and no mechanistic inference can be drawn from these data alone.

A different nominal association pattern was observed for *IL1B* rs16944. In the adjusted analysis, the CC genotype showed a nominal association with higher odds of T2DM under the recessive model. This pattern is consistent with the known role of IL-1β in inflammatory signaling and its reported involvement in β-cell injury, impaired insulin secretion, and insulin resistance [32]. In contrast to rs11556218, the nominal association for rs16944 was observed primarily in homozygous carriers, suggesting a possible recessive pattern that requires confirmation in independent studies. Previous studies examining this polymorphism in relation to glucose metabolism have reported inconsistent results, with some cohorts showing associations with glycemic traits or diabetes risk, and others showing no clear association [21,23,33]. The available evidence suggests that the contribution of rs16944 to T2DM risk may be modest and influenced by population-specific factors.

The clinical and metabolic profile of the T2DM group was broadly consistent with the expected characteristics of the disease. Compared with controls, participants with T2DM showed the expected clustering of cardiometabolic abnormalities, including higher BMI, WHR, triglycerides, fasting plasma glucose, and blood pressure, lower HDL cholesterol, and a higher frequency of family history of diabetes. This overall pattern was consistent with the established clinical profile of T2DM [30,31]. Total cholesterol and LDL cholesterol levels were also lower in the T2DM group. Although information on lipid-lowering medication use was not systematically collected, statins are routinely prescribed to eligible patients with T2DM in our clinical setting. Therefore, the lower total cholesterol and LDL cholesterol levels may reflect the effects of lipid-lowering therapy rather than the untreated lipid profile of the T2DM group and should be interpreted with caution. The effect estimates remained broadly similar after adjustment for BMI, WHR, and family history of diabetes, indicating that they were not materially altered by the selected covariates. VIF values indicated no meaningful multicollinearity between BMI and WHR.

Several points should be considered when interpreting the present findings. First, because this was an analytical cross-sectional observational case-control study, the results should be interpreted as associations rather than evidence of temporal or causal relationships. The observed associations may reflect direct effects of the investigated variants, linkage with other functional loci in the same genomic region, or interaction with unmeasured factors such as diet, physical activity, or other inflammatory mediators. In addition, HbA1c testing and an oral glucose tolerance test were not performed in the control group. Although controls had no previous diagnosis of diabetes and fasting plasma glucose levels <100 mg/dL at enrollment, dysglycemia detectable only by HbA1c or an oral glucose tolerance test could not be excluded. Consequently, some participants with undiagnosed prediabetes or, less likely, diabetes may have been misclassified as controls. This potential misclassification may have reduced the contrast between the two groups and biased the estimated genotype-T2DM associations toward the null. Second, the study was restricted to Kinh Vietnamese individuals. Although this reduces ethnic heterogeneity and mimimise the possibility of residual population stratification, it may also limit the generalizability of the findings to populations with different genetic backgrounds or environmental exposures. In addition, participants were recruited using convenience sampling at a tertiary referral hospital. Participants with T2DM attending this setting may have had longer disease duration, more complex glycemic management, or a greater burden of comorbidities and complications than those managed in primary care or community settings. Therefore, selection bias cannot be excluded, and the findings may not be fully generalizable to the broader community-based Kinh Vietnamese population. Information on lipid-lowering medication use, including statin type, dose, and treatment duration, was not systematically collected. Therefore, the influence of such treatment on the between-group differences in total cholesterol and LDL cholesterol could not be quantified. Third, although the sample size met the prespecified power requirements, some genotype categories were relatively uncommon, which may have reduced precision in certain models and contributed to wide confidence intervals. In addition, although several nominal associations were observed, none met the Bonferroni-corrected significance threshold. The findings should therefore be interpreted as exploratory and require confirmation in independent populations. Finally, circulating IL-1β and IL-16 concentrations were not measured, and no functional experiments were performed. The present results should therefore be viewed as statistical genetic associations rather than direct evidence of a biological mechanism. This distinction is important, since circulating cytokine levels may not fully capture inflammatory processes occurring in disease-relevant tissues. IL-1β has been implicated in the pancreatic islet microenvironment and local β-cell dysfunction [33], whereas IL-16 is more closely linked to immune cell recruitment and activation within inflamed tissues [22]. Accordingly, the absence of cytokine profiling does not exclude tissue-level effects, but it does limit biological interpretation.

Despite these limitations, this study provides preliminary population-specific evidence of nominal associations between *IL16* rs11556218, *IL1B* rs16944, and T2DM in Kinh Vietnamese adults. The different patterns observed across the two loci raise the possibility that distinct inflammatory pathways may be related to diabetes susceptibility in different ways, although these findings require independent replication. Further research in larger cohorts, preferably with longitudinal and functional approaches, is needed to determine whether these associations are reproducible and to clarify their biological significance in this population.

## Conclusion

In conclusion, *IL16* rs11556218 and *IL1B* rs16944 showed nominal associations with T2DM in Kinh Vietnamese adults under different genetic models. However, none of these associations remained statistically significant after Bonferroni correction for multiple testing. These exploratory findings require confirmation in larger independent studies, ideally with functional investigation, before any clinical implications can be drawn.

## Supporting information

S1 Data Full data. (XLSX)
