# Supplementary material for: Association of IL16 rs11556218 and IL1B rs16944 polymorphisms with type 2 diabetes in the Kinh Vietnamese population
Source: PLoS One. 2026 Sep 28;21(9):e0358570. doi: 10.1371/journal.pone.0358570 (PMC13618935; doi:10.1371/journal.pone.0358570)
Supplement: Supplementary file 1 [file pone.0358570.s002.docx]

**Review Comments for the manuscript titled “ASSOCIATION OF IL16 rs11556218 AND IL1B rs16944 POLYMORPHISMS WITH TYPE 2 DIABETES IN THE KINH VIETNAMESE POPULATION”**

**Comments to Authors**

The manuscript investigates the association of IL16 rs11556218 and IL1B rs16944 polymorphisms with type 2 diabetes mellitus in a Kinh Vietnamese population. The topic is relevant and scientifically important; however, several methodological and reporting issues require major revision before the manuscript can be considered for publication.

**Major Comments**

**Comment 1**: The adjusted analyses include only BMI and WHR as covariates. Given the significant differences in family history of diabetes and other metabolic characteristics between groups, the rationale for selecting only BMI and WHR should be justified. The authors should consider additional multivariable analyses, including family history and other clinically relevant covariates, to assess the robustness of the reported associations.

**Comment 2**: The rationale for simultaneously adjusting for BMI and WHR should be clarified, as both variables are related measures of adiposity and may lead to over-adjustment in genetic association analyses.

**Comment 3**: The manuscript does not address multiple testing despite evaluating two polymorphisms under several inheritance models. The authors should perform an appropriate correction for multiple comparisons or justify why such a correction was not applied.

**Comment 4**: The authors should discuss the possibility of residual population stratification and clarify whether any measures were taken to minimise this bias.

**Comment 5**: Controls were classified as non-diabetic based on fasting plasma glucose <100 mg/dL and absence of a prior diabetes diagnosis. Since HbA1c values are not reported for controls and do not appear to have been used in the eligibility criteria, some individuals with undiagnosed diabetes or prediabetes may have been misclassified. Please clarify whether HbA1c testing was performed in controls and discuss the potential impact of misclassification bias.

**Minor Comments**

**Comment 6**: Please clarify whether the study should be described as a case-control design rather than a cross-sectional study.

**Comment 7**: Consider including HWE p-values directly in the genotype frequency table.

**Comments to Editor**

The manuscript may be considered for publication after satisfactory revision addressing the

above comments.
