# Supplementary material for: Association of IL16 rs11556218 and IL1B rs16944 polymorphisms with type 2 diabetes in the Kinh Vietnamese population
Source: PLoS One. 2026 Sep 28;21(9):e0358570. doi: 10.1371/journal.pone.0358570 (PMC13618935; doi:10.1371/journal.pone.0358570)
Supplement: Peer review history

# Peer review: Decision Letter 0

**DOI:** 10.1371/journal.pone.0358570.r001

**Authors:** Milad Khorasani

15 Jul 2026

Dear Dr. Duc Do,

Thank you for submitting your manuscript to PLOS ONE. After careful consideration, we feel that it has merit but does not fully meet PLOS ONE’s publication criteria as it currently stands. Therefore, we invite you to submit a revised version of the manuscript that addresses the points raised during the review process.

Please submit your revised manuscript by Sep 13 2026 11:59PM. If you will need more time than this to complete your revisions, please reply to this message or contact the journal office at plosone@plos.org. When you're ready to submit your revision, log on to https://www.editorialmanager.com/pone/ and select the 'Submissions Needing Revision' folder to locate your manuscript file.

- A letter that responds to each point raised by the academic editor and reviewer(s). You should upload this letter as a separate file labeled 'Response to Reviewers'.
- A marked-up copy of your manuscript that highlights changes made to the original version. You should upload this as a separate file labeled 'Revised Manuscript with Track Changes'.
- An unmarked version of your revised paper without tracked changes. You should upload this as a separate file labeled 'Manuscript'.

If you would like to make changes to your financial disclosure, please include your updated statement in your cover letter. Guidelines for resubmitting your figure files are available below the reviewer comments at the end of this letter.

If applicable, we recommend that you deposit your laboratory protocols in protocols.io to enhance the reproducibility of your results. Protocols.io assigns your protocol its own identifier (DOI) so that it can be cited independently in the future. For instructions see: https://journals.plos.org/plosone/s/submission-guidelines#loc-laboratory-protocols. Additionally, PLOS ONE offers an option for publishing peer-reviewed Lab Protocol articles, which describe protocols hosted on protocols.io. Read more information on sharing protocols at https://plos.org/protocols?utm_medium=editorial-email&utm_source=authorletters&utm_campaign=protocols.

As the corresponding author, your ORCID iD is verified in the submission system and will appear in the published article. PLOS supports the use of ORCID, and we encourage all coauthors to register for an ORCID iD and use it as well. Please encourage your coauthors to verify their ORCID iD within the submission system before final acceptance, as unverified ORCID iDs will not appear in the published article. *Only*  the individual author can complete the verification step; PLOS staff *cannot*  verify ORCID iDs on behalf of authors.

We look forward to receiving your revised manuscript.

Kind regards,

Milad Khorasani, PhD

Academic Editor

PLOS One

Journal Requirements:

When submitting your revision, we need you to address these additional requirements.

1. Please ensure that your manuscript meets PLOS ONE's style requirements, including those for file naming. The PLOS ONE style templates can be found at

https://journals.plos.org/plosone/s/file?id=wjVg/PLOSOne_formatting_sample_main_body.pdf and

https://journals.plos.org/plosone/s/file?id=ba62/PLOSOne_formatting_sample_title_authors_affiliations.pdf.

2. In the online submission form, you indicated that [Insert text from online submission form here].

All PLOS journals now require all data underlying the findings described in their manuscript to be freely available to other researchers, either 1. In a public repository, 2. Within the manuscript itself, or 3. Uploaded as supplementary information.

This policy applies to all data except where public deposition would breach compliance with the protocol approved by your research ethics board. If your data cannot be made publicly available for ethical or legal reasons (e.g., public availability would compromise patient privacy), please explain your reasons on resubmission and your exemption request will be escalated for approval.

3.Your ethics statement should only appear in the Methods section of your manuscript. If your ethics statement is written in any section besides the Methods, please delete it from any other section.

4. Please include captions for your Supporting Information files at the end of your manuscript, and update any in-text citations to match accordingly. Please see our Supporting Information guidelines for more information: http://journals.plos.org/plosone/s/supporting-information.

5. If the reviewer comments include a recommendation to cite specific previously published works, please review and evaluate these publications to determine whether they are relevant and should be cited. There is no requirement to cite these works unless the editor has indicated otherwise.

[Note: HTML markup is below. Please do not edit.]

Reviewers' comments:

Reviewer's Responses to Questions

**Comments to the Author**

1. Is the manuscript technically sound, and do the data support the conclusions?

Reviewer #1: Partly

Reviewer #2: Yes

**********

2. Has the statistical analysis been performed appropriately and rigorously?

Reviewer #1: No

Reviewer #2: Yes

**********

3. Have the authors made all data underlying the findings in their manuscript fully available?

The PLOS Data policy

Reviewer #1: Yes

Reviewer #2: No

**********

4. Is the manuscript presented in an intelligible fashion and written in standard English?

Reviewer #1: Yes

Reviewer #2: Yes

**********

Reviewer #1: Comments to Authors

The manuscript investigates the association of IL16 rs11556218 and IL1B rs16944 polymorphisms with type 2 diabetes mellitus in a Kinh Vietnamese population. The topic is relevant and scientifically important; however, several methodological and reporting issues require major revision before the manuscript can be considered for publication.

Major Comments

Comment 1: The adjusted analyses include only BMI and WHR as covariates. Given the significant differences in family history of diabetes and other metabolic characteristics between groups, the rationale for selecting only BMI and WHR should be justified. The authors should consider additional multivariable analyses, including family history and other clinically relevant covariates, to assess the robustness of the reported associations.

Comment 2: The rationale for simultaneously adjusting for BMI and WHR should be clarified, as both variables are related measures of adiposity and may lead to over-adjustment in genetic association analyses.

Comment 3: The manuscript does not address multiple testing despite evaluating two polymorphisms under several inheritance models. The authors should perform an appropriate correction for multiple comparisons or justify why such a correction was not applied.

Comment 4: The authors should discuss the possibility of residual population stratification and clarify whether any measures were taken to minimise this bias.

Comment 5: Controls were classified as non-diabetic based on fasting plasma glucose <100 mg/dL and absence of a prior diabetes diagnosis. Since HbA1c values are not reported for controls and do not appear to have been used in the eligibility criteria, some individuals with undiagnosed diabetes or prediabetes may have been misclassified. Please clarify whether HbA1c testing was performed in controls and discuss the potential impact of misclassification bias.

Minor Comments

Comment 6: Please clarify whether the study should be described as a case-control design rather than a cross-sectional study.

Comment 7: Consider including HWE p-values directly in the genotype frequency table.

Comments to Editor

The manuscript may be considered for publication after satisfactory revision addressing the

above comments.

Reviewer #2: This paper addresses a relevant topic and provides novel population-specific data for the Kinh Vietnamese. The novel population data on Kinh Vietnamese individuals and the adequate sample size, along with power calculations, are the strengths of this article.

1. The authors adjusted only for BMI and WHR, yet Table 1 reveals numerous additional significant differences between cases and controls, including triglycerides, HDL cholesterol, blood pressure, and family history of diabetes. While BMI and WHR are appropriate adiposity measures, the rationale for excluding other potential confounders, particularly family history (a strong, well-established T2DM risk factor) and metabolic traits integral to disease pathophysiology, is not adequately justified. If these variables are considered mediators on the causal pathway between the genetic variants and T2DM, their exclusion from the model may be appropriate; however, this should be explicitly stated.

2. The authors tested two SNPs under four genetic models (8 primary tests). No correction for multiple comparisons (e.g., Bonferroni) is applied. While exploratory, this should be acknowledged as a limitation. The authors should either apply a correction and reinterpret results, or explicitly state that these are exploratory findings.

3. Controls were defined as having FPG < 100 mg/dL at enrollment. Were Oral Glucose Tolerance Test or HbA1c performed to exclude prediabetes? If not, some controls may have undiagnosed dysglycemia (impaired fasting glucose or prediabetes), which could bias results toward the null. This should be acknowledged as a limitation.

4. Abstract (lines 41) states the TG genotype was significant in the codominant model (adjusted OR = 0.54). However, Results (lines 230-231) show the crude OR for TG is 0.56, but the unadjusted P value in the abstract seems inconsistent with the full results. Please verify all abstract figures match the full text.

5. The Kinh are the majority ethnic group, but Vietnam has substantial ethnic diversity.

The authors should clarify how participants were recruited. Were they recruited consecutively, randomly, or via convenience sampling? Was the hospital a tertiary referral center? If so, cases may be more severe, potentially influencing results.

6. Line 195-196: VIF < 2 is acceptable, but why were only BMI and WHR included? This should be clarified earlier.

7. Total cholesterol and LDL are lower in cases. This is unusual in T2DM, which typically presents with dyslipidemia (high LDL, low HDL). Is this because patients are on statins? The authors don't mention medication use. This could be a major confounder and should be addressed.

**********

what does this mean?). If published, this will include your full peer review and any attached files.

If you choose “no”, your identity will remain anonymous but your review may still be made public.

**Do you want your identity to be public for this peer review?**  For information about this choice, including consent withdrawal, please see our Privacy Policy

Reviewer #1: No

Reviewer #2: No

**********

[NOTE: If reviewer comments were submitted as an attachment file, they will be attached to this email and accessible via the submission site. Please log into your account, locate the manuscript record, and check for the action link "View Attachments". If this link does not appear, there are no attachment files.]

To ensure your figures meet our technical requirements, please review our figure guidelines: https://journals.plos.org/plosone/s/figures

You may also use PLOS’s free figure tool, NAAS, to help you prepare publication quality figures: https://journals.plos.org/plosone/s/figures#loc-tools-for-figure-preparation.

NAAS will assess whether your figures meet our technical requirements by comparing each figure against our figure specifications.

Attachment Submitted filename: Review Comments 20-06-26.docx

# Author comment: Author response to Decision Letter 1

**DOI:** 10.1371/journal.pone.0358570.r002

4 Aug 2026

Response to the Editor and Reviewers

Manuscript title: Association of IL16 rs11556218 and IL1B rs16944 polymorphisms with type 2 diabetes in the Kinh Vietnamese population

Manuscript ID: PONE-D-26-21867

Dear Academic Editor and Reviewers,

We sincerely thank the Academic Editor and the Reviewers for their careful evaluation of our manuscript and for their constructive comments. We have carefully considered each comment and revised the manuscript accordingly. Our point-by-point responses are provided below. The Reviewers’ comments are reproduced in bold, followed by our responses. All revisions are visible in the marked-up version labeled “Revised Manuscript with Track Changes.” The line numbers cited in our responses refer to the clean revised version labeled “Manuscript.”

We hope that the revisions have adequately addressed the concerns raised and improved the clarity and rigor of the manuscript.

Response to Reviewer #1

We sincerely thank you for the careful assessment of our manuscript and for the constructive comments and suggestions. We have addressed each comment as detailed below.

Major Comments

Comment 1: The adjusted analyses include only BMI and WHR as covariates. Given the significant differences in family history of diabetes and other metabolic characteristics between groups, the rationale for selecting only BMI and WHR should be justified. The authors should consider additional multivariable analyses, including family history and other clinically relevant covariates, to assess the robustness of the reported associations.

Response:

Thank you for this helpful suggestion. We agree that family history of diabetes is a well-established risk factor for T2DM and should be considered in the adjusted analyses. Accordingly, we repeated the adjusted analyses by including family history of diabetes as an additional covariate, together with BMI and WHR. The effect estimates remained largely unchanged after this additional adjustment, indicating that they were not materially affected by the inclusion of family history of diabetes.

We did not include the other metabolic characteristics solely because they differed significantly between the T2DM and control groups, as a between-group difference does not necessarily indicate confounding. Fasting plasma glucose directly reflects glycemic status and is closely related to the classification of participants as cases or controls. Blood pressure, lipid parameters, and serum creatinine may reflect metabolic abnormalities associated with T2DM, diabetes-related consequences, or the effects of treatment after diagnosis. Adjusting for these variables could therefore result in over-adjustment and potentially attenuate the genotype–T2DM associations under investigation.

Changes in the manuscript:

Family history of diabetes was added as a covariate in the adjusted analyses in the Statistical Analysis section. The adjusted ORs, 95% CIs, and P values were updated in Tables 3 and 4 and in the corresponding Results subsections for IL16 rs11556218 and IL1B rs16944. The Abstract, Discussion, and Conclusion were also revised to reflect the additional adjustment and the updated estimates.

Comment 2: The rationale for simultaneously adjusting for BMI and WHR should be clarified, as both variables are related measures of adiposity and may lead to over-adjustment in genetic association analyses.

Response:

Thank you for raising this important point. BMI and WHR were included because they reflect different aspects of adiposity: BMI indicates overall adiposity, whereas WHR reflects abdominal fat distribution. Both are associated with the risk of type 2 diabetes and may therefore influence the estimated associations between the polymorphisms and T2DM.

We assessed multicollinearity between BMI and WHR before including them simultaneously in the adjusted analyses. As shown in Table 5, both variables had a tolerance value of 0.719 and a VIF of 1.39, indicating no evidence of meaningful multicollinearity. We therefore retained both variables in the adjusted analyses to account for both overall adiposity and abdominal fat distribution.

Changes in the manuscript:

The Statistical Analysis section was revised to explain that BMI and WHR were included because they reflect overall adiposity and abdominal fat distribution, respectively. The wording describing the VIF assessment was also clarified, and Table 5 was retitled “Assessment of multicollinearity between BMI and WHR.”

Comment 3: The manuscript does not address multiple testing despite evaluating two polymorphisms under several inheritance models. The authors should perform an appropriate correction for multiple comparisons or justify why such a correction was not applied.

Response:

Thank you for raising this important issue. We applied a Bonferroni correction to account for the eight primary adjusted association tests arising from the evaluation of two polymorphisms under four genetic models. Accordingly, the corrected significance threshold was set at P < 0.00625 (0.05/8). None of the associations met this threshold. The smallest nominal P values were 0.0081 for the log-additive model of rs11556218 and 0.0089 for the recessive model of rs16944, both of which exceeded the Bonferroni-corrected threshold. The original P values have been retained for transparency, but findings with nominal P values <0.05 are now described as nominal associations and interpreted as exploratory. The interpretation of the findings has been revised throughout the manuscript accordingly.

Changes in the manuscript:

The Bonferroni correction and the corrected significance threshold were added to the Statistical Analysis section. The Abstract, Results, Discussion, and Conclusion were revised to distinguish nominal associations from findings meeting the corrected threshold. The footnotes to Tables 3 and 4 were also revised to state that no adjusted association remained statistically significant after Bonferroni correction.

Comment 4: The authors should discuss the possibility of residual population stratification and clarify whether any measures were taken to minimise this bias.

Response:

Thank you for this suggestion. The possibility of residual population stratification cannot be totally excluded, and we tried to minimise this bias by recruiting only Kinh Vietnamese in this study.

Changes in the manuscript:

This point was added to the Discussion part in line 383.

Comment 5: Controls were classified as non-diabetic based on fasting plasma glucose <100 mg/dL and absence of a prior diabetes diagnosis. Since HbA1c values are not reported for controls and do not appear to have been used in the eligibility criteria, some individuals with undiagnosed diabetes or prediabetes may have been misclassified. Please clarify whether HbA1c testing was performed in controls and discuss the potential impact of misclassification bias.

Response:

We appreciate this suggestion. HbA1c testing was not performed in the control group. Controls were selected based on the absence of a previous diagnosis of diabetes and a fasting plasma glucose level <100 mg/dL at enrollment, as stated in the Materials and Methods section. Although this criterion excluded individuals whose fasting plasma glucose levels met the thresholds for impaired fasting glucose or diabetes at enrollment, it could not exclude dysglycemia detectable only by HbA1c or an oral glucose tolerance test. We therefore acknowledge that some individuals with undiagnosed prediabetes or, less likely, diabetes may have been included in the control group. This potential misclassification may have reduced the contrast between the two groups and biased the estimated genotype–T2DM associations toward the null.

Changes in the manuscript:

The Clinical and Biochemical Assessment subsection was revised to state that HbA1c was measured only in participants with T2DM and was unavailable for controls. The absence of HbA1c and oral glucose tolerance testing in controls, together with the potential impact of misclassification bias, was added as a limitation in the Discussion.

Minor Comments

Comment 6: Please clarify whether the study should be described as a case-control design rather than a cross-sectional study.

Response:

We appreciate this comment and the opportunity to clarify the study design. We have clarified the design as an analytical cross-sectional study because all participants were recruited during the same defined study period, and their diabetes status, clinical and biochemical characteristics, and genetic data were assessed at a single point in time at enrollment. Participants were classified into T2DM and non-T2DM comparison groups according to their diabetes status at the time of assessment. No longitudinal follow-up or retrospective ascertainment of exposure was performed.

Although equal numbers of participants were included in the two groups, this reflected the planned sampling allocation rather than individual matching. Participants with T2DM were not individually matched to those without T2DM according to age, sex, or other characteristics. The study design and the formation of the comparison groups have been clarified in the Materials and Methods section.

Changes in the manuscript:

The study design was specified as an analytical cross-sectional study in the Abstract, the Study Design and Participants subsection, the Discussion, and the Conclusion.

Comment 7: Consider including HWE p-values directly in the genotype frequency table.

Response:

Thank you for this helpful suggestion. The HWE P values for both polymorphisms have been added directly to the genotype frequency table. HWE was assessed in the control group using an exact test, with P values of 0.63 for IL16 rs11556218 and 0.15 for IL1B rs16944. The separate supplementary table reporting these results was removed to avoid duplication.

Changes in the manuscript:

A column presenting the control-group HWE P values was added to Table 2. The citation in the Results subsection “Genotype distributions and Hardy–Weinberg equilibrium” was changed from “Supplementary Table” to “Table 2,” and the separate supplementary HWE table was removed.

Reviewer #2: We sincerely thank you for the thoughtful evaluation of our manuscript and for recognizing the relevance and novelty of our population-specific data, as well as the adequacy of the sample size and power calculations. We greatly appreciate the constructive comments, which have helped us further strengthen the manuscript. Our point-by-point responses are provided below.

Comment 1: The authors adjusted only for BMI and WHR, yet Table 1 reveals numerous additional significant differences between cases and controls, including triglycerides, HDL cholesterol, blood pressure, and family history of diabetes. While BMI and WHR are appropriate adiposity measures, the rationale for excluding other potential confounders, particularly family history (a strong, well-established T2DM risk factor) and metabolic traits integral to disease pathophysiology, is not adequately justified. If these variables are considered mediators on the causal pathway between the genetic variants and T2DM, their exclusion from the model may be appropriate; however, this should be explicitly stated.

Response:

We appreciate this thoughtful comment. We agree that family history of diabetes is a well-established risk factor for T2DM and should be considered in the adjusted analyses. We therefore repeated the adjusted analyses with family history of diabetes included as an additional covariate, together with BMI and WHR. The effect estimates remained largely unchanged after this additional adjustment, indicating that they were not materially affected by the inclusion of family history of diabetes.

The remaining metabolic variables were not included solely on the basis of their significant differences between the T2DM and control groups, as a between-group difference does not necessarily indicate confounding. Fasting plasma glucose directly reflects glycemic status and is closely related to the classification of participants as having or not having T2DM. Blood pressure and lipid parameters may represent concurrent metabolic abnormalities, lie downstream of the disease process, or be influenced by treatment after diagnosis. We therefore did not consider these variables appropriate baseline confounders for the associations between the polymorphisms and T2DM. Adjusting for them could result in over-adjustment and potentially attenuate the associations under investigation. This rationale has been clarified in the revised manuscript.

Changes in the manuscript:

Family history of diabetes was added as a covariate in the adjusted analyses, and the rationale for covariate selection was clarified in the Statistical Analysis section. The adjusted ORs, 95% CIs, and P values were updated in Tables 3 and 4 and in the corresponding Results subsections. The Abstract, Discussion, and Conclusion were also revised to reflect the additional adjustment and updated estimates.

Comment 2: The authors tested two SNPs under four genetic models (8 primary tests). No correction for multiple comparisons (e.g., Bonferroni) is applied. While exploratory, this should be acknowledged as a limitation. The authors should either apply a correction and reinterpret results, or explicitly state that these are exploratory findings.

Response:

We appreciate this comment. As suggested, we applied a Bonferroni correction to the eight primary two-sided adjusted association tests corresponding to two SNPs evaluated under four genetic models. This yielded a corrected significance threshold of P < 0.00625 (0.05/8). Although several associations had nominal P values below 0.05, none remained statistically significant after correction. We have retained the original P values for transparency but revised the manuscript to describe these findings as nominal associations, interpret them as exploratory, and emphasize the need for independent replication.

Changes in the manuscript:

The Bonferroni correction was described in the Statistical Analysis section. The interpretation of the findings was revised in the Abstract, Results, Discussion, and Conclusion, and explanatory footnotes were added to Tables 3 and 4.

Comment 3: Controls were defined as having FPG < 100 mg/dL at enrollment. Were an oral glucose tolerance test or HbA1c performed to exclude prediabetes? If not, some controls may have undiagnosed dysglycemia (impaired fasting glucose or prediabetes), which could bias results toward the null. This should be acknowledged as a limitation.

Response:

We appreciate this comment. Neither HbA1c testing nor an oral glucose tolerance test was performed in the control group. Controls were selected based on the absence of a previous diagnosis of diabetes and a fasting plasma glucose level <100 mg/dL at enrollment. Although this criterion excluded individuals whose fasting plasma glucose levels met the thresholds for impaired fasting glucose or diabetes at enrollment, it could not exclude dysglycemia detectable only by HbA1c or an oral glucose tolerance test. We therefore acknowledge that some individuals with undiagnosed prediabetes or, less likely, diabetes may have been included in the control group. This potential misclassification may have reduced the contrast between the two groups and biased the estimated genotype–T2DM associations toward the null.

Changes in the manuscript:

The Clinical and Biochemical Assessment subsection was revised to clarify that HbA1c was measured only in participants with T2DM and was unavailable for controls. The absence of HbA1c and oral glucose tolerance testing in controls, together with the potential impact of misclassification bias, was added as a limitation in the Discussion.

Comment 4: Abstract (lines 41) states the TG genotype was significant in the codominant model (adjusted OR = 0.54). However, Results (lines 230-231) show the crude OR for TG is 0.56, but the unadjusted P value in the abstract seems inconsistent with the full results. Please verify all abstract figures match the full text.

Response:

We appreciate this careful observation. We verified all numerical results reported in the Abstract against the corresponding crude and adjusted analyses in the full text and Tables 3 and 4. The Abstract has been corrected to distinguish the crude and adjusted estimates and to ensure consistency with the revised analyses. Specifically, the adjusted codominant model is now reported with an overall P value of 0.026, while the genotype-specific comparison of TG versus TT yielded an adjusted OR of 0.56 (95% CI: 0.32–0.98). The results for the dominant and log-additive models of IL16 rs11556218 and the recessive model of IL1B rs16944 were also updated to match Tables 3 and 4. These findings are now described as nominal associations because none met the Bonferroni-corrected significance threshold.

Changes in the manuscript:

The adjusted ORs, 95% CIs, and P values in the Results portion of the Abstract were corrected and updated to match Tables 3 and 4. The Abstract was also revised to identify these findings as nominal associations that did not meet the Bonferroni-corrected significance threshold.

Comment 5: The Kinh are the majority ethnic group, but Vietnam has substantial ethnic diversity. The authors should clarify how participants were recruited. Were they recruited consecutively, randomly, or via convenience sampling? Was the hospital a tertiary referral center? If so, cases may be more severe, potentially influencing results.

Response:

Thank you for highlighting this issue. Participants were recruited using convenience sampling at the Outpatient Department of Cho Ray Hospital. Cho Ray Hospital is a tertiary referral hospital that receives patients from Ho Chi Minh City and other provinces in southern Vietnam. Because the study specifically aimed to investigate the two polymorphisms in the Kinh Vietnamese population, only Kinh Vietnamese individuals were included.

We acknowledge that convenience sampling at a tertiary referral hospital may have introduced selection bias. Participants with T2DM attending this setting may have had longer disease duration, more complex glycemic management, or a greater burden of comorbidities and complications than those managed in primary care or community settings. Therefore, the study sample may not be fully representative of the broader community-based Kinh Vietnamese population. The recruitment method and hospital setting have been clarified in the Materials and Methods section, and the potential selection bias and limited generalizability have been acknowledged in the Discussion.

Changes in the manuscript:

The use of convenience sampling and the tertiary referral setting of Cho Ray Hospital were specified in the Study Design and Participants subsection. The potential for selection bias and limited generalizability to the broader community-based Kinh Vietnamese population were added as limitations in the Discussion.

Comment 6: Line 195-196: VIF < 2 is acceptable, but why were only BMI and WHR included? This should be clarified earlier.

Response:

We appreciate this comment. Family history of diabetes has now been added as an additional covariate in the adjusted analyses. The rationale for selecting BMI and WHR has also been clarified earlier in the Statistical Analysis section. BMI and WHR were included because they reflect different aspects of adiposity: BMI reflects overall adiposity, whereas WHR reflects abdominal fat distribution. As shown in Table 5, the VIF value of 1.39 indicated no evidence of meaningful multicollinearity between BMI and WHR.

Changes in the manuscript:

The Statistical Analysis section was revised to specify adjustment for BMI, WHR, and family history of diabetes and to explain why BMI and WHR were both included. Table 5 was retitled to specify that the multicollinearity assessment concerned BMI and WHR.

Comment 7: Total cholesterol and LDL are lower in cases. This is unusual in T2DM, which typically presents with dyslipidemia (high LDL, low HDL). Is this because patients are on statins? The authors don't mention medication use. This could be a major confounder and should be addressed.

Response:

We appreciate this important observation. Information on statin use was not systematically collected in this study; therefore, we cannot determine the proportion of participants receiving statins or formally assess their effect on lipid levels. In our clinical setting, statin therapy is routinely prescribed to eligible patients with T2DM as part of guideline-based care. It is therefore plausible that lipid-lowering treatment contributed to the lower total cholesterol and LDL cholesterol levels observed in the T2DM group.

We acknowledge that the absence of detailed medication data prevents us from distinguishing the effect of statin therapy from the underlying lipid profile of the participants. The lipid findings should therefore be interpreted with caution. This explanation and limitation have been added to the Discussion.

Changes in the manuscript:

The possible contribution of statin therapy to the lower total cholesterol and LDL cholesterol levels in the T2DM group was discussed in the paragraph describing the participants’ clinical and metabolic profiles. The absence of systematically collected information on lipid-lowering medication use and its impact on interpretation of the lipid findings was also acknowledged in the limitations paragraph.

Attachment Submitted filename: Response to Reviewers.docx

# Peer review: Decision Letter 1

**DOI:** 10.1371/journal.pone.0358570.r003

**Authors:** Milad Khorasani

2 Sep 2026

<p>ASSOCIATION OF IL16 rs11556218 AND IL1B rs16944 POLYMORPHISMS WITH TYPE 2 DIABETES IN THE KINH VIETNAMESE POPULATION

PONE-D-26-21867R1

Dear Dr. Duc Do,

We’re pleased to inform you that your manuscript has been judged scientifically suitable for publication and will be formally accepted for publication once it meets all outstanding technical requirements.

Within one week, you’ll receive an e-mail detailing the required amendments. When these have been addressed, you’ll receive a formal acceptance letter and your manuscript will be scheduled for publication.

An invoice will be generated when your article is formally accepted. Please note, if your institution has a publishing partnership with PLOS and your article meets the relevant criteria, all or part of your publication costs will be covered. Please make sure your user information is up-to-date by logging into Editorial Manager at Editorial Manager® and clicking the ‘Update My Information' link at the top of the page. For questions related to billing, please contact billing support.

If your institution or institutions have a press office, please notify them about your upcoming paper to help maximize its impact. If they’ll be preparing press materials, please inform our press team as soon as possible -- no later than 48 hours after receiving the formal acceptance. Your manuscript will remain under strict press embargo until 2 pm Eastern Time on the date of publication. For more information, please contact onepress@plos.org.

Kind regards,

Milad Khorasani, PhD

Academic Editor

PLOS One

Additional Editor Comments (optional):

Reviewers' comments:

Reviewer's Responses to Questions

**Comments to the Author**

Reviewer #2: All comments have been addressed

Reviewer #3: (No Response)

**********

2. Is the manuscript technically sound, and do the data support the conclusions?

Reviewer #2: Yes

Reviewer #3: Yes

**********

3. Has the statistical analysis been performed appropriately and rigorously?

Reviewer #2: Yes

Reviewer #3: Yes

**********

4. Have the authors made all data underlying the findings in their manuscript fully available?

The PLOS Data policy

Reviewer #2: No

Reviewer #3: Yes

**********

5. Is the manuscript presented in an intelligible fashion and written in standard English?

Reviewer #2: Yes

Reviewer #3: Yes

**********

Reviewer #2: Thank you, author, for submitting a revised manuscript and for the detailed responses to the concerns raised during the initial review. I have carefully evaluated the revisions and am pleased to see that the author has addressed the major issues thoroughly and thoughtfully. I also note that the author has corrected minor discrepancies between the abstract and the results and has expanded the discussion to provide more context on potential biological mechanisms and population-specific considerations. These improvements enhance the clarity and completeness of the manuscript.

Reviewer #3: The authors have addressed the major concerns raised in the previous round, and the revised manuscript has been substantially improved. In particular, the authors have appropriately addressed multiple testing by applying a Bonferroni correction, added family history of diabetes to the adjusted analyses, clarified the rationale for covariate selection, and acknowledged the limitations related to control classification, convenience sampling, and lack of systematic information on statin use.

However, I recommend a final careful check of the revised manuscript before publication.

The interpretation of the genetic associations should be fully consistent with the Bonferroni correction. The authors state in their response that none of the associations remained statistically significant after correction and that the findings should therefore be considered nominal and exploratory. However, the Abstract provided in the revised manuscript still uses expressions such as “significant differences” and “remained significantly associated.” These statements should be revised to consistently describe the findings as nominal associations or exploratory findings, as appropriate.

The Abstract should be carefully cross-checked against the revised Tables and Results to ensure that all adjusted ORs, 95% CIs, and P values are identical and that the distinction between nominal P values and Bonferroni-corrected statistical significance is clearly maintained.

The Methods and Abstract should also be checked for consistency regarding the covariates included in the adjusted analyses. The revised response indicates that BMI, WHR, and family history of diabetes were included, whereas the Abstract currently mentions adjustment only for BMI and WHR.

The terminology used throughout the manuscript should be standardized. In particular, the terms “statistically significant association,” “nominal association,” and “exploratory finding” should be used consistently and in accordance with the Bonferroni-corrected threshold.

Subject to these minor corrections and a final consistency check of the manuscript, I consider the study technically sound and the revised analyses generally appropriate.

**********

what does this mean?). If published, this will include your full peer review and any attached files.

If you choose “no”, your identity will remain anonymous but your review may still be made public.

**Do you want your identity to be public for this peer review?**  For information about this choice, including consent withdrawal, please see our Privacy Policy

Reviewer #2: No

Reviewer #3: No

**********

# Editor report: Acceptance letter

**DOI:** 10.1371/journal.pone.0358570.r004

**Authors:** Milad Khorasani

PONE-D-26-21867R1

PLOS One

Dear Dr. Do,

I'm pleased to inform you that your manuscript has been deemed suitable for publication in PLOS One. Congratulations! Your manuscript is now being handed over to our production team.

At this stage, our production department will prepare your paper for publication. This includes ensuring the following:

* All references, tables, and figures are properly cited

* All relevant supporting information is included in the manuscript submission,

* There are no issues that prevent the paper from being properly typeset

You will receive further instructions from the production team, including instructions on how to review your proof when it is ready. Please keep in mind that we are working through a large volume of accepted articles, so please give us a few days to review your paper and let you know the next and final steps.

Lastly, if your institution or institutions have a press office, please let them know about your upcoming paper now to help maximize its impact. If they'll be preparing press materials, please inform our press team within the next 48 hours. Your manuscript will remain under strict press embargo until 2 pm Eastern Time on the date of publication. For more information, please contact onepress@plos.org.

You will receive an invoice from PLOS for your publication fee after your manuscript has reached the completed accept phase. If you receive an email requesting payment before acceptance or for any other service, this may be a phishing scheme. Learn how to identify phishing emails and protect your accounts at https://explore.plos.org/phishing.

If we can help with anything else, please email us at customercare@plos.org.

Thank you for submitting your work to PLOS One and supporting open access.

Kind regards,

PLOS One Editorial Office Staff

on behalf of

Dr. Milad Khorasani

Academic Editor

PLOS One
